# The Role of Oxidative Stress in Atopic Dermatitis and Chronic Urticaria

**DOI:** 10.3390/antiox11081590

**Published:** 2022-08-16

**Authors:** Sabina Galiniak, Mateusz Mołoń, Marek Biesiadecki, Agnieszka Bożek, Marta Rachel

**Affiliations:** 1 Institute of Medical Sciences, Medical College of Rzeszow University, Rzeszow University, Warzywna 1a, 35-310 Rzeszow, Poland; 2 Department of Biology, Institute of Biology and Biotechnology, Rzeszow University, Zelwerowicza 4, 35-601 Rzeszow, Poland; 3 Department of Allergology and Cystic Fibrosis, Provincial Hospital No. 2 in Rzeszow, Lwowska 60, 35-301 Rzeszow, Poland

**Keywords:** atopic dermatitis, chronic urticaria, lipid peroxidation, protein oxidation

## Abstract

Atopic dermatitis (AD) and chronic urticaria (CU) are common skin diseases with an increasing prevalence and pathogenesis that are not fully understood. Emerging evidence suggests that oxidative stress plays a role in AD and CU. The aim of the single-center cross-sectional study was to compare markers of oxidative stress in 21 patients with AD, and 19 CU patients. The products of protein oxidation, total antioxidant capacity (TAC), and markers of lipid peroxidation were estimated in the serum. AD patients had a higher level of advanced protein oxidation products and a lower level of thiol groups than healthy participants. However, CU patients had statistically higher levels of AOPP and 3-nitrotyrosine than healthy subjects. The level of thiol groups and serum TAC decreased significantly in patients with CU. There was no difference in serum concentration of lipid peroxidation products, Amadori products, ratio of reduced to oxidized glutathione, and ability of albumin to binding cobalt between AD or CU patients compared to healthy subjects. We found a moderate positive significant correlation between AOPP and age in patients with AD. In patients with CU, TAC was negatively correlated with age. These results may shed light on the etiopathogenesis of AD or CU, and confirm an oxidative burden in these patients. Furthermore, our study could be useful in developing new therapeutic methods that include using antioxidants in dermatological diseases.

## 1. Introduction

Atopic dermatitis (AD) and chronic urticaria (CU) are common skin diseases with an increasing prevalence, now affecting not only developed countries but also low-income countries [1,2]. AD is a chronic condition characterized by pruritic skin lesions associated with epithelial barrier dysfunction with immunologic involvement that manifests as dry and itchy skin. AD occurs in 10–20% of children and about 3% of adults [3,4]. AD is also a disorder of elderly people, which is likely underestimated even if increasingly recognized [5]. The clinical presentation of AD is highly variable, depending on the age and severity of the disease. Acute lesions are characterized by pruritic papules with erythema, xerosis, and serous exudation [1]. CU is an autoimmune condition, where it is mediated by IgG autoantibodies, and an “autoallergic” condition, where it is mediated by the so-called highly cytokinergic IgE [6]. Moreover, CU is defined by the repeated appearance of itchy hives, angioedema, or both, for 6 weeks or more [7]. CU occurred in approximately 2.1% of children and 1.1% of adolescents [8]. The pathogenesis of AD and CU is not fully understood; however, significant progress has been made in recent years. The pathogenesis of AD and CU is not fully understood; however, significant progress has been made in recent years. Oxidative stress, defined as an imbalance between the generation of reactive oxygen species (ROS) and the mechanisms of their elimination, accompany many diseases, including psoriasis, asthma, cystic fibrosis, neurodegenerative diseases, and cancer [9,10,11,12,13]. Oxidative stress leads to lipid peroxidation, protein oxidation, and DNA damage, thus inducing several physiological dysfunctions and loss of biological activity [14]. Emerging evidence suggests that oxidative stress plays a role in AD and CU [15,16]. Furthermore, oxidative stress is positively correlated with disease activity [17]. It is related to the fact that the skin harbors a wide range of enzymatic and nonenzymatic compounds that act as antioxidants or oxidant-degrading molecules [18]. 

The aim of the study was to compare markers of oxidative stress in the serum of patients with AD and CU. Second, we tried to assess the correlation between oxidative stress markers and clinical parameters of patients with AD or CU. Here we are the first to report on the level of products of protein oxidation and 4-hydroxy-2-nonenal in serum of patients with AD or CU.

## 2. Materials and Methods

### 2.1. Ethical Issues

The Bioethics Committee of the University of Rzeszow, Poland, approved the protocol for this study (2022/006, date: 12 January 2021). All procedures performed in studies involving human participants were in accordance with the ethical standards of the institutional and/or national research committee and with the Declaration of Helsinki of 1964 and its subsequent amendments or comparable ethical standards. Written informed consent was obtained from all participants or, if participants were under 16, from a parent and/or legal guardian.

### 2.2. Study Group

Twenty-one AD patients and 19 CU patients who were admitted to the local dermatology and allergology clinic of the Provincial Hospital No. 2, Rzeszow, Poland, were enrolled in a single-center cross-sectional study. Study participants were recruited between April 2021 and April 2022.

The study involved patients with AD, diagnosed according to Hanifn and Rajka criteria, and patients with CU, diagnosed according to practice guidelines [19,20]. The diagnosis was made by a specialist in the field of dermatology. Patients were enrolled in the study when AD or CU was present for at least 6 months before participating.

Exclusion criteria were also as follows: failure to sign informed consent by the patient, presence of other inflammatory skin diseases (e.g., Netherton’s syndrome, T-cell cutaneous lymphoma, extensive contact dermatitis, chronic solar dermatitis), any surgical, medical (e.g., asthma, uncontrolled hypertension, heart failure, diabetes, congenital and genetic defects, obesity, immunodeficiency), smoking habit, a psychiatric or physical condition that the dermatologist believes could put the patient at risk if they participated in this study. Patients were treated with topical steroids, antihistamines, and emollients which did not interfere with systemic inflammation and oxidative stress.

At the same time, 14 healthy people were included in the control group. The control group consisted of people who volunteered for the study, met the inclusion criteria, and tested negative on the skin prick test (inclusion criteria: no history of allergy, AD, CU, and other chronic diseases, normal hematological results, non-smoking, no use of any antioxidant vitamins). The participants had not taken any medications 30 days before the study. All participants had anthropometric measurements. BMI was calculated as kg/m^2^.

### 2.3. Study Group Section

The study involved 21 patients with AD, 19 with CU, and 14 healthy controls, 39% of whom were women. Patient characteristics are summarized in Table 1. 

The participants in the studied group were identical in terms of age and BMI. There were also no differences in the levels of white blood cells between the study groups. The highest percentage of eosinophilia was found in the group of people with AD, where it was statistically higher than in healthy subjects (*p* < 0.001) and participants with UC (*p* < 0.01). Furthermore, in people with AD, total IgE increased significantly compared to healthy people (*p* < 0.05). Furthermore, CRP was elevated in the AD group compared to the CU group (*p* < 0.05). Interestingly, total IgE was high in people with CU, but was not statistically elevated compared to the rest of the study groups.

### 2.4. Materials

All basic chemical reagents were purchased from Sigma-Aldrich (Poznan, Poland) unless otherwise indicated. All reagents used were of analytical reagent grade. The 3-nitrotyrosine enzyme-linked immunosorbent assay (ELISA) kit was bought from Immunodiagnostik AG (K7829, Bensheim, Germany). The 4-hydroxy-2-nonenal (4-HNE) ELISA kit was purchased from Wuhan Fine Biotech Co., Ltd. (EU0187, Wuhan, China). All measurements were performed on a Tecan Infinite 200 PRO multimode reader (Tecan Group Ltd.; Männedorf, Switzerland). Measurements were made in triplicate unless otherwise indicated.

### 2.5. Blood Collection

Blood samples were collected, centrifugated, and stored according to the standard procedure.

### 2.6. Blood Counts, Serum Analysis, and Allergy Test

Morphology of blood cells was performed using an automated laboratory analyzer (Siemens Healthineers, Erlangen, Germany). C-reactive protein (CRP) was assayed using the dry chemistry immunological method on a VITROS 250 analyzer (Ortho Clinical Diagnostics, Johnson and Johnson, New Brunswick, NJ, USA). Total IgE was assayed by the using enzyme immunoassay (VIDAS bioMérieux, S.A, Lyon, France) according to the manufacturer’s protocol. Both groups underwent inhaled allergen skin prick testing with inhaled allergens (Skin prick tests—BASIC SET inhaled Allergopharma, Reinbek, Germany).

### 2.7. Biochemical Procedures

#### 2.7.1. Protein

The method of Lowry et al. was used to estimate protein concentration [21].

#### 2.7.2. Advanced Oxidation Protein Products 

Advanced oxidation protein products (AOPP) were determined using the method of Witko-Sarsat et al. [22]. AOPP level was expressed in nmol chloramine-T equivalents/mg protein.

#### 2.7.3. Thiol Group

The Ellman method was used to determine the content of thiol groups [23]. The thiol group content was expressed in mmol/L.

#### 2.7.4. 3-Nitrotyrosine

The 3-nitrotyrosine level was determined with the 3-nitrotyrosine ELISA kit (Immundiagnostik AG) according to the manufacturer’s protocol and expressed as nM/mg protein.

#### 2.7.5. Total Antioxidant Capacity with ABTS

The total antioxidant capacity of serum with ABTS^•^ was determined by using the method of Re et al. [24] and expressed in Trolox equivalents (μmol TE/L).

#### 2.7.6. Total Antioxidant Capacity with FRAP

Ferric reducing antioxidant power assay (FRAP) was determined colorimetrically by measuring the ferric reducing capacity of serum samples and expressed in Trolox equivalents (μmol TE/L) [25].

#### 2.7.7. Malondialdehyde

The Yagi method was used to determine the level of malondialdehyde (MDA) expressed as μmol/L [26]. The results were calculated using an absorption coefficient for MDA of 1.56 × 105 M^−^^1^ cm^−^^1^.

#### 2.7.8. 4-Hydroxy-2-Nonenal

The 4-hydroxy-2-nonenal (4-HNE) concentration was determined with the 4-HNE ELISA kit (Wuhan Fine Biotech Co., Ltd., Wuhan, China) according to the manufacturer’s protocol and expressed as pg/mL.

#### 2.7.9. GSH/GSSG Ratio

The GSH/GSSG ratio was estimated according to the recently described method by Kovalčíková et al. [27].

#### 2.7.10. Amadori Product

The level of the Amadori product was determined using the method of Johnson et al. [28]. The Amadori products were calculated using an extinction coefficient of 12,640 M^−^^1^ cm^−^^1^ for monoformazan [29]. Measurements were made in duplicate.

#### 2.7.11. Albumin Cobalt Binding

Albumin cobalt binding levels were estimated by Bar-Or’s method and expressed in absorbance units (ABSU) [30].

### 2.8. Statistical Analysis

Data are presented as arithmetic mean values and standard deviations. Statistical significance of differences was analyzed using the Kruskal-Wallis test. Spearman’s rank correlation coefficient analysis was used to estimate the correlation between oxidative stress markers and patients’ clinical parameters, assuming linear dependence. Statistical analysis was estimated using the STATISTICA software package (version 13.3, StatSoft Inc., 2017, Tulsa, OK, USA).

## 3. Results

**Figure 1:**
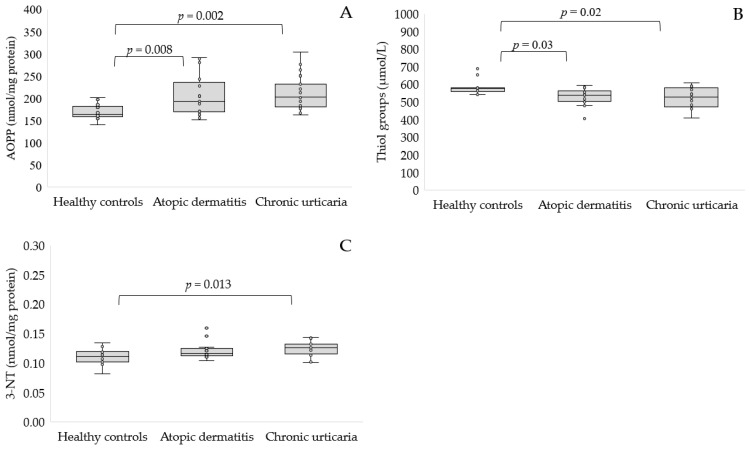
Concentration of AOPP (**A**), thiol groups (**B**) and 3-nitrotyrosine (**C**) in serum of patients with AD or CU as compared to the control group.

Proteins are particularly susceptible to ROS damage. AOPP is recognized as a novel marker of oxidative stress formed during oxidative stress by the interaction between proteins and oxidants. In our study, AOPP levels increased significantly in people with AD (208.38 ± 46.29 nmol/mg protein, *p* < 0.01) or CU patients compared to healthy people (210.8 ± 39.36 vs. 169.23 ± 17.39 nmol/mg protein, *p* < 0.01, Figure 1A). Protein thiol oxidation can occur due to ROS reacting directly with cysteine or methionine residues. Our study indicates that the concentration of the thiol groups was significantly lower in AD (530.6 ± 46.07 mmol/L, *p* < 0.05) and CU participants compared to control subjects (523.33 ± 55.82 vs. 581.97 ± 40.5 mmol/L, *p* < 0.05, Figure 1B). Although the side chain of several amino acids is susceptible to oxidative modification, tyrosine residues are one of the preferred targets of single-electron oxidants, leading to the formation of 3-nitrotyrosine. CU patients had statistically higher levels of 3-nitrotyrosine than healthy subjects (*p* < 0.05, Figure 1C). The concentration of this marker was similar in AD patients and controls. 

**Figure 2:**
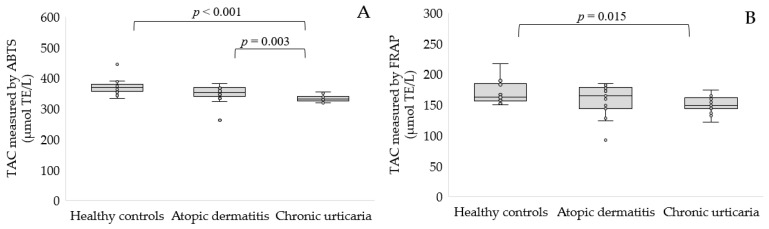
TAC level determined by ABTS^•^ (**A**) and FRAP (**B**) in the serum of patients with AD or CU as compared to the control group.

Subsequently, we estimated the total antioxidant capacity of the serum of the studied groups using two methods. We demonstrated, using the ABTS^•^ method, a significantly reduced serum TAC in patients with AD (*p* < 0.01) and CU (*p* < 0.001) patients compared to healthy volunteers (Figure 2A). The second FRAP method confirmed a significantly reduced TAC of the serum of participants with CU compared to healthy controls (150.75 ± 13.05 vs. 170.16 ± 19.06 μmol TE/L, *p* < 0.05, Figure 2B).

Table 2 presents the results of estimating the remaining markers of oxidative stress.

Two of the well-studied markers of lipid peroxidation are malondialdehyde (MDA) and 4-hydroxy-2-nonenal. We found elevated levels of MDA in the serum of people with CU compared to healthy people (*p* = 0.023), while the 4-HNE level did not differ between these groups. There was no difference in the level of these lipid peroxidation markers between AD and healthy controls. The reduced (GSH)-to-oxidized (GSSG) glutathione ratio represents a dynamic balance between oxidants and antioxidants in serum. There was no statistical difference in the serum GSH to GSSG ratio between the study groups.

Furthermore, reducing sugar can react non-enzymatically with amino acid residues of protein to form Amadori products that generate ROS in the presence of transition metals and molecular oxygen. The concentration of Amadori products was similar in the studied groups. Moreover, we used the albumin cobalt binding test to compare the cobalt binding affinity for albumin as a modification of the specific cobalt binding site by ROS. However, we found no difference in the ability of albumin to bind cobalt ions between healthy subjects and patients with AD or CU.

The dependence between markers of oxidative stress level and general characteristics, as well as hematological and serum biochemical parameters, was estimated using the Spearman correlation and presented in Table 3. 

We found a moderate positive significant correlation between AOPP and age (R = 0.533, *p* = 0.011) in patients with AD. Furthermore, negative correlations between TAC and BMI were found in the AD group. The TAC measured by ABTS^•^ was negatively correlated with WBC, while the TAC measured by FRAP was negatively correlated with CRP in participants with AD. In patients with CU, TAC measured by ABTS^•^ was negatively correlated with age (R = −0.624, *p* = 0.007). In addition, a moderate positive significant correlation between MDA and EOS was estimated in CU individuals. We did not find any additional associations between other parameters analyzed.

## 4. Discussion

Determining circulating levels of oxidative stress markers is an area of increasing interest, as is the detection of inflammatory markers in many diseases, including dermatological disease. Recent studies focusing on inflammatory markers report that basal IgE and D-dimer levels could be used to assess treatment response, including recombinant humanized monoclonal IgG antibodies [31]. Different markers are currently available to estimate oxidative stress, but none of them can be considered ideal. 

To our knowledge, the level of AOPP and 3-nitrotyrosine in the serum of AD patients has not yet been studied. We have shown that AD patients have significantly increased AOPP levels compared to healthy controls, while the 3-nitrotyrosine level was similar in these studied groups. Upon oxidative stress, excess ROS reacts with proteins, leading to their oxidative damage, including modification of amino acid residues and prosthetic groups in complex proteins, the breaking of the polypeptide chain, and the formation of cross-links between polypeptide chains. These structural changes result in an increase or loss of biological activity [32]. The level of protein carbonyl moieties, the next marker of protein oxidation, was elevated and correlated directly with the severity of AD [33]. Additionally, the concentrations of nitrite and nitrate were lower, and the protein-bound nitrotyrosine content was significantly increased in the skin lesions in NC/Nga mice, an animal model for human AD that could suggest that reactive nitrogen species are involved in the pathogenesis of AD [34]. A recent study by Iizumi et al. indicated that the 6-nitrotryptophan content of the Ig light chains could be a new biomarker to detect the early stage of AD, which confirmed elevated protein nitration [35]. In vitro AD model keratinocytes exhibited elevated ROS levels and accumulated more DNA damage than control cells after induction of oxidative stress, which leads to reduced survival and may contribute to the formation of lesions in AD [36]. Elevated levels of oxidation products can be caused by increased O_2_^−^ production, which may participate in the pathogenesis of this skin disease [37]. Furthermore, AD patients had significantly increased myeloperoxidase activity, a leukocyte-derived enzyme that catalyzes the formation of several ROS [38]. On the other hand, patients with CU had significantly higher levels of AOPP and 3-nitrotyrosine compared to healthy controls, which indicates oxidative damage to serum proteins. Similarly, AOPP levels in the spontaneous form of CU were elevated compared to healthy participants (*p* < 0.001) [15]. Surprisingly, patients with acute urticaria had a significantly higher level of carbonyl protein in serum (*p* < 0.001) according to the control group; however, these results may be due to the small size of the study group [39]. We used two methods to determine the serum TAC. In the method with ABTS^●^ and FRAP, we did not notice a reduced TAC in AD compared to healthy subjects. However, both methods showed a lower TAC in the serum of people with CU compared to healthy people. Additionally, the ABTS^●^ method indicated a significantly reduced TAC in patients with CU compared to AD. Interpretation of TAC changes in biological samples, including serum, depends on the methodology used. Regarding the FRAP test, its major components in human samples are uric acid, which represents approximately 60% of the final value, a-tocopherol, bilirubin, and ascorbic acid, while it does not determine the thiol group containing antioxidants such as GSH and albumin. In addition to the compounds measured by the FRAP method, the ABTS test also measures albumin, which is approximately half the final value in human samples, while the proportion of uric acid in the final ABTS value is lower than that of the FRAP test [40]. The TAC measured by the new automated colorimetric method developed by Erel was similar in infants with AD concerning healthy controls [41]. Furthermore, TAC measured using the oxygen radical absorbance capacity method was also decreased in AD compared to healthy controls (10.1 ± 0.15 vs. 13.2 ± 0.21 mmol TE/L, *p* < 0.001) [38]. The median plasma level of TAC in the spontaneous CU decreased significantly compared to that of the control group (*p* = 0.001), which is in line with our results. At the same time, plasma total oxidant status increased significantly in children with a spontaneous form of CU compared to healthy participants (*p* = 0.003) [17]. It is worth noting that the activity of antioxidant enzymes in CU may be decreased, indicating insufficient mechanisms for the elimination of ROS [42,43]. The redox buffer GSH is characterized by the thiol group (-SH) of cysteine. GSH is converted to its dimer GSSG after reducing the target molecules. Therefore, the GSH/GSSG ratio indicates the oxidative state [44]. Moreover, thiol-disulphide balance plays an important role in health and disease. In our study, there were no differences in the GSH/GSSG ratio between AD patients and controls. However, we found significantly lower thiol groups in AD than in controls. However, in the AD group, the serum disulphide and the ratios of disulphide/native thiol and disulphide/total thiol were significantly lower than in the healthy controls in the study by Uysal et al. [45]. Similarly, a decrease in GSH concentration was reported among AD patients [46]. The mean concentrations of native thiol and total thiol were lower (*p* = 0.012), while the mean disulfide concentration was significantly higher in infants with AD than in the control group (*p* = 0.025) [41]. There were no differences in the GSH/GSSG ratio in serum of patients with CU compared to healthy controls. However, patients with CU had a significantly lower level of thiol groups with respect to healthy controls, which is consistent with a previous report [47]. Akbas et al. reported no statistical differences in patients with acute urticaria compared to the control group in the levels of native thiols, disulphides, and total thiols. However, for patients with chronic CU, there was an increase in levels of native thiols, disulphides, and total thiols, and the ratio of thiol/disulphide in favor of disulphide [48]. In previous studies, a significantly higher level of MDA (*p* < 0.001), and a lower level of antioxidant enzymes and antioxidants in patients with AD compared to healthy controls were reported [46,49]. In our study, we did not find any differences between the AD group and healthy controls in the level of MDA and 4-HNE, which belong to lipid peroxidation products. Similar to our results, increased but not statistically significant levels of MDA and 4-HNE were found in exhaled breath condensate from AD patients [50]. Furthermore, an elevated level of lipid hydroperoxides was found in children with AD [38]. We found an increase in MDA level in the serum of patients with CU, while the 4-HNE concentration of 4-HNE was similar to that of healthy subjects. No differences in MDA were observed in 25 adult patients with an idiopathic form of CU compared to healthy controls [42]. Similarly, plasma and erythrocyte MDA levels were similar in CU with and without positive response to the autologous serum skin test, as well as in healthy females [51]. However, platelet MDA levels were significantly elevated in spontaneous CU compared to healthy controls [43]. So far, the level of 4-HNE in the course of CU has not been determined. Amadori products are stable intermediates in the glycation process, the nonenzymatic reaction process of proteins and sugars, which may be exacerbated by oxidative stress [52]. The glucose-derived Amadori product reacts with itself or primary amines and undergoes further reactions to form advanced glycation end products (AGEs), a heterogeneous group of bioactive compounds [53]. Corneocyte AGEs level was increased in the AD group (*p* = 0.002) compared to healthy participants. Furthermore, a higher level of corneocyte AGEs was observed in severe AD than in the milder form of AD. However, no significant difference in serum AGE levels in patients with AD and healthy controls, which is consistent with our results for glycation products [54]. However, urinary concentrations of pentosidine, one of the AGEs, were significantly higher in patients with acute exacerbation of AD than in healthy controls and patients with stable AD [55]. On the other hand, a decrease in serum AGE concentration in the spontaneous form of CU was reported in the study by Grzanka et al., which is different from our results, as we did not find any difference in the level of glycation products [56]. This may be due to the lower albumin concentration reported in this study, while our patients had protein levels to the control. However, in a previous study, no differences in AGEs in serum of patients with the spontaneous form of CU and healthy participants were also reported [15]. Subsequently, we used the albumin cobalt binding test to estimate the oxidative damage of albumin, which has altered the binding capacity to bind transition metals such as cobalt or copper in its N-terminus region. It is produced during ischemia or oxidative stress when a series of chemical reactions occur that alter the N-terminus of albumin, thus making it unable to bind to those metals [57]. However, we did not notice changes in the level of binding to albumin in both AD and CU, which may indicate that this protein is not damaged by oxidative stress. However, patients with acute urticaria or CU showed a reduced ability of serum albumin to bind cobalt ions compared to healthy controls, which could result from the inflammatory response induced by oxidative stress [47,58,59]. Similarly, patients with psoriasis revealed a lower ability of albumin to bind ions than healthy controls, which might be produced through an adaptive response to chronic hypoxia and oxidative stress, which might play a role in the systemic inflammation seen in psoriasis [60].

Oxidative stress markers often correlate with the clinical parameters of the patients, including age, BMI, inflammatory markers, or disease duration under normal conditions and in many diseases [61,62,63]. In our study, several correlations were determined, mainly related to TAC, negatively correlated with BMI, percentage of white blood cells, and CRP concentration in AD, and negatively correlated with age in patients with CU. Furthermore, AOPP was positively correlated with age among AD patients and MDA with a percentage of blood eosinophils among people with CU. The urinary marker of oxidation of a DNA, 8-hydroxy-2′-deoxyguanosine, was not correlated with age, disease duration, or disease severity in AD [64]. The TAC values showed a statistically significant negative correlation with the duration of the disease in patients with the spontaneous form of CU [17]. However, there was no significant correlation between age, total IgE levels, eosinophil count and TAC, and total levels of oxidant status levels in children with acute urticaria [65]. No significant correlations between AOPP and AGE and duration of urticaria were also determined in the study by Nettis et al. [15]. Given the association of oxidative stress with AD and CU, it is worthwhile to consider incorporating strategies to reduce oxidative stress in managing these conditions. This can be accomplished in many ways, including lowering ROS production and increasing antioxidant status, reducing the intensity of inflammation and proinflammatory cytokine release. Moreover, patients should avoid environmental, physical, and psychological triggers to achieve prolonged remissions; and apply emollients to maintain the intact skin barrier. The practical recommendation would be a combination of anti-inflammatory agents, immunomodulating drugs, skin emollients, and antioxidants [66].

Our study presents interesting and new information on oxidative stress markers in patients with AD and CU. One of the strengths of our study was that the studied groups consisted of Caucasian volunteers living in the same city, sharing similar environmental conditions and cultural habits, which limits the influence of various factors on the studied markers. It is worth mentioning that the strength of our study is also the inclusion of a variety of antioxidative and oxidative stress biomarkers, some of which have not been measured before in these patients. However, some limitations should be mentioned. First of all, our study considers a small number of patients. In addition, it is a single-center study. Finally, we did not analyze the amount of antioxidants consumed in the diet.

## 5. Conclusions

In conclusion, these results may shed light on the etiopathogenesis of AD, as well as CU, and confirm an oxidative burden in these patients. Furthermore, our study could be useful in developing new therapeutic methods that include using antioxidants in dermatological diseases. Further research should focus on evaluating the drugs, including corticosteroids and calcineurin inhibitors, related to the level of oxidative stress markers.

## Figures and Tables

**Table 1:** Characteristics of patients at enrollment ^a^.

		Healthy Controls	AD	CU
Sex (F/M)		6/8	8/13	7/12
Age (years)	mean ± SD	25.71 ± 13.18	25.73 ± 10.24	29.06 ± 16.51
	range	6–46	7–44	5–46
BMI	mean ± SD	21.79 ± 1.9	21.64 ± 3.48	22.56 ± 3.7
	range	17–25	14–25	14–25
Clinical laboratory markers				
WBC (10^3^/µL)	mean ± SD	6.79 ± 1.59	7.72 ± 2.53	7.02 ± 1.62
	range	4.1–10	4.2–13.8	4.2–10.1
EOS (%)	mean ± SD	1.11 ± 0.69	7.54 ± 5.52 ^A^***^,B^**	2.95 ± 3.26
	range	0.3–2.3	1.2–15.5	0.6–12.2
Total IgE (kU/L) (reference range: adults and children <10 years: <25 kU/L—negative; 25–100 kU/L—doubtful; >100 kU/L—positive)	mean ± SD	19.97 ± 15.05	763.23 ± 811.77 ^A^*	155.97 ± 206.41
	range	1.4–51.3	0–2989.4	0–653.5
CRP (mg/L)	mean ± SD	2.33 ± 1.42	2.48 ± 3.81 ^B^*	3.97 ± 2.43
	range	0.3–4.3	0.5–17.7	0.5–11

^a^ AD—atopic dermatitis, CU—chronic urticaria, BMI—body mass index, WBC—white blood cells, EOS—eosinophils, CRP—C-reactive protein; differences between means were analyzed using the Kruskal-Wallis test using Statistica software (version 13.3, StatSoft Inc., 2017, Tulsa, OK, USA); ^A^ when compared AD with HC, ^B^ when compared AD with CU, * *p* < 0.05, ** *p* < 0.01, *** *p* < 0.001.

**Table 2:** Markers of oxidative stress in healthy controls and patients with AD or CU ^a^.

		Healthy Controls	AD	CU
MDA (μmol/L)	mean ± SD	3.16 ± 0.3	3.55 ± 0.64	3.67 ± 0.62 ^A,^*
	range	2.77–3.72	2.91–5.49	2.91–5.24
4-HNE (pg/mL)	mean ± SD	340.57 ± 85.86	354.39 ± 127.67	377.95 ± 124.74
	range	209.28–533.04	186.77–686.28	224.89–641.83
GSH/GSSG ratio	mean ± SD	3.06 ± 0.27	3.05 ± 0.24	2.91 ± 0.29
	range	2.63–3.63	2.58–3.36	2.38–3.45
Amadori product (nmol/mg protein)	mean ± SD	1609.73 ± 230.09	1651.77 ± 231.45	1695.54 ± 239.3
	range	1132.86–1816.82	1366.55–2407.55	1366.51–2227.36
Albumin cobalt binding (ABSU)	mean ± SD	0.28 ± 0.04	0.3 ± 0.03	0.28 ± 0.04
	range	0.22–0.34	0.23–0.35	0.21–0.34

^a^ AD—atopic dermatitis, CU—chronic urticarial, 4-HNE—4-hydroxy-2-nonenal, MDA—malondialdehyde, GSH/GSSG ratio—reduced (GSH)-to-oxidized (GSSG) glutathione ratio; differences between means were analyzed using Kruskal-Wallis test using Statistica software (version 13.3, StatSoft Inc., 2017, Tulsa, OK, USA); ^A^ when compared CU with HC, * *p* < 0.05.

**Table 3:** Spearman’s rank correlation coefficients and *p* values ^a^.

			Age	BMI	WBC	EOS	CRP	Total IgE
Atopic dermatitis	AOPP	*R*	0.533	0.255	0.212	0.041	−0.235	−0.021
		*p*	0.011	0.251	0.342	0.857	0.304	0.924
	Thiol groups	*R*	0.207	0.226	−0.041	0.042	−0.314	−0.086
		*p*	0.354	0.311	0.855	0.851	0.165	0.703
	3-Nitrotyrosine	*R*	−0.055	−0.053	−0.118	0.051	−0.05	−0.092
		*p*	0.806	0.814	0.599	0.815	0.831	0.685
	TAC measured by ABTS^•^	*R*	0.306	−0.405	−0.476	0.555	−0.411	0.206
		*p*	0.176	0.036	0.028	0.265	0.072	0.371
	TAC measured by FRAP	*R*	0.322	−0.531	0.296	0.037	−0.451	0.072
		*p*	0.154	0.028	0.192	0.871	0.046	0.754
	MDA	*R*	−0.063	−0.18	−0.226	0.06	0.364	0.182
		*p*	0.779	0.422	0.311	0.789	0.105	0.418
	4-HNE	*R*	−0.196	−0.085	0.008	0.102	0.096	−0.199
		*p*	0.384	0.707	0.972	0.648	0.678	0.374
	GSH/GSSG ratio	*R*	0.262	0.301	−0.049	0.054	−0.285	0.308
		*p*	0.239	0.174	0.828	0.813	0.051	0.164
	Amadori products	*R*	0.378	0.34	0.162	−0.052	−0.075	−0.061
		*p*	0.082	0.121	0.473	0.818	0.748	0.785
	Albumin cobalt binding	*R*	0.271	0.314	−0.07	−0.119	0.003	0.077
		*p*	0.223	0.154	0.756	0.597	0.988	0.73
Chronic urticaria	AOPP	*R*	0.282	0.051	−0.386	−0.235	−0.004	0.001
		*p*	0.272	0.844	0.112	0.348	0.988	0.996
	Thiol groups	*R*	0.077	−0.12	−0.149	−0.265	0.1	−0.448
		*p*	0.767	0.646	0.553	0.287	0.691	0.07
	3-Nitrotyrosine	*R*	0.066	−0.335	−0.155	−0.042	0.385	0.053
		*p*	0.799	0.187	0.537	0.867	0.114	0.841
	TAC measured by ABTS^•^	*R*	−0.624	−0.387	0.073	0.005	−0.344	0.224
		*p*	0.007	0.125	0.772	0.984	0.162	0.386
	TAC measured by FRAP	*R*	−0.271	−0.368	−0.012	0.248	−0.277	0.082
		*p*	0.277	0.132	0.96	0.306	0.279	0.747
	MDA	*R*	−0.318	−0.218	0.053	0.574	−0.252	0.071
		*p*	0.213	0.4	0.835	0.013	0.313	0.786
	4-HNE	*R*	0.211	0.002	0.042	−0.195	0.244	−0.255
		*p*	0.416	0.998	0.867	0.436	0.329	0.323
	GSH/GSSG ratio	*R*	0.442	0.335	0.129	0.371	0.365	−0.085
		*p*	0.075	0.187	0.609	0.13	0.137	0.747
	Amadori products	*R*	−0.137	−0.111	−0.277	−0.311	−0.223	0.139
		*p*	0.601	0.673	0.265	0.208	0.373	0.593
	Albumin cobalt binding	*R*	0.455	0.404	−0.455	−0.459	0.325	−0.257
		*p*	0.065	0.107	0.057	0.056	0.188	0.318

^a^ BMI—body mass index, WBC—white blood cells, EOS—eosinophils, CRP—C-reactive protein.

## Data Availability

All data generated or analysed during this study are included in this published article.
